# Supplementary material for: Dysfunction in IGF2R Pathway and Associated Perturbations in Autophagy and WNT Processes in Beckwith–Wiedemann Syndrome Cell Lines
Source: Int J Mol Sci. 2024 Mar 22;25(7):3586. doi: 10.3390/ijms25073586 (PMC11011696; doi:10.3390/ijms25073586)
Supplement: Supplementary file 1 [file ijms-25-03586-s001.zip › ijms-2876696-supplementary.pdf]

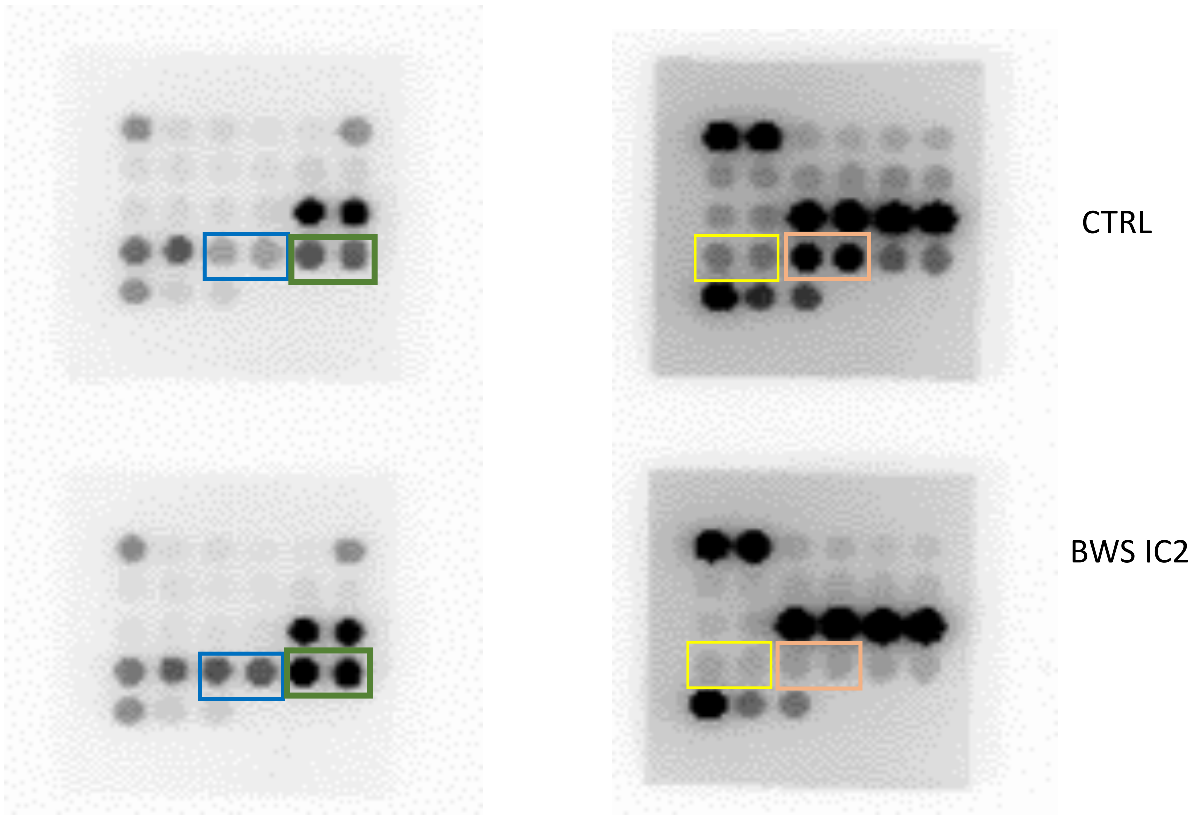

Supplemental Figure S1. Analysis of the phosphorylation pattern of components of the PI3K and MAPK pathways by PathScan EGFR signaling antibody array Kit in BWS IC2 and CTRL LCLs. Phosphorylation of MEK1/2 (Ser221) and MEK1/2 (Ser217/221) are highlighted in blue and green, respectively. Phosphorylation of Akt (Thr308) and Akt (Ser473) are highlighted in yellow and orange, respectively.

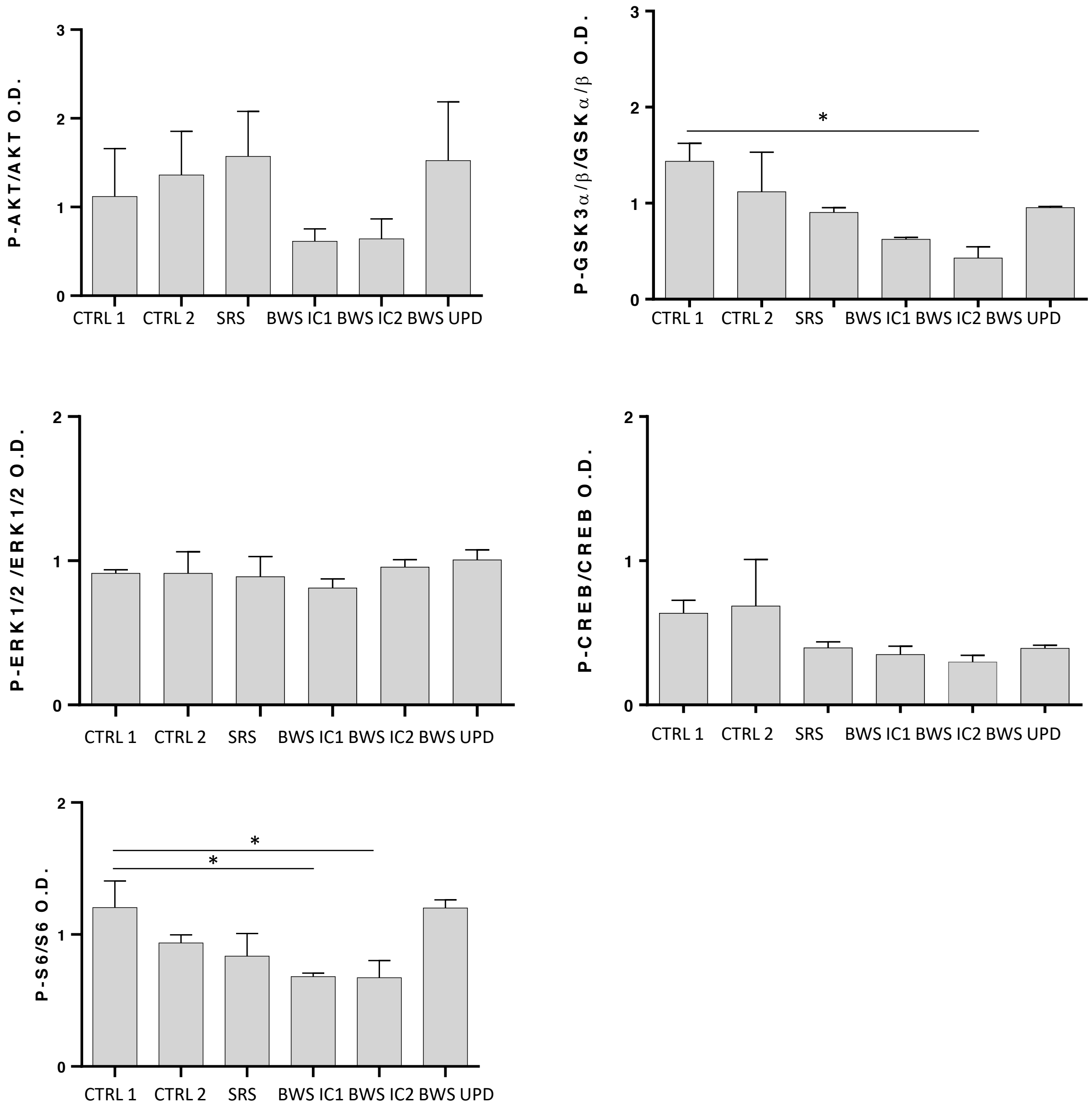

Supplemental Figure S2. Relative intensity (Optical density: O.D.) by densitometric analysis was evaluated and expressed as ratio of phospho/total proteins. Data represent the mean ± SEM of 3 experiments (two way-ANOVA, *<0.05)

Table S1. Panel of genes of the WNT pathway analyzed by nCounter approach

GENE	Accession	Log2 fold change	P-value	BH.p.value	Pathway annotation
TP53	NM_000546.2	0.632	2.81e-05	0.00446	KEGG WNT Annotation
PLAUR	NM_001005376.1	-2.16	0.000288	0.0229	Proteolysis
CUL1	NM_003592.2	0.196	0.000798	0.0377	KEGG WNT Annotation
PRKCB	NM_212535.1	-1.23	0.00102	0.0377	KEGG WNT Annotation
FRAT1	NM_005479.3	1.83	0.00146	0.0377	Canonical Wnt Pathway, KEGG WNT Annotation
AXIN1	NM_181050.1	0.265	0.00149	0.0377	Canonical Wnt Pathway, KEGG WNT Annotation, WNT Signaling Negative Regulation
MAPK9	NM_139068.2	0.51	0.00166	0.0377	KEGG WNT Annotation
CEBPD	NM_005195.3	1.58	0.0027	0.0481	Transcription Factors
DKK4	NM_014420.2	-1.2	0.00272	0.0481	KEGG WNT Annotation
MAPK10	NM_002753.2	-6.57	0.00526	0.079	KEGG WNT Annotation
CREBBP	NM_001079846.1	0.547	0.00573	0.079	KEGG WNT Annotation
PITX2	NM_000325.5	3.94	0.00649	0.079	Transcription Factors, WNT Signaling Target Genes
RBX1	NM_014248.2	-0.521	0.00682	0.079	KEGG WNT Annotation
MMP9	NM_004994.2	2.09	0.00722	0.079	Calcium Binding and Signaling, Development & Differentiation, Migration, Proteolysis
FBXW11	NM_033645.2	0.313	0.00745	0.079	KEGG WNT Annotation, WNT Signaling Negative Regulation
WNT10A	NM_025216.2	-2.25	0.00837	0.0832	Calcium Binding and Signaling, Canonical Wnt Pathway, KEGG WNT Annotation
GSK3A	NM_019884.2	0.167	0.0168	0.157	Canonical Wnt Pathway

GDNF	NM_000514.2	-4.12	0.0222	0.185	Development & Differentiation, Migration
SMAD2	NM_005901.5	0.278	0.0268	0.206	EMTMetastasis, KEGG WNT Annotation
SMAD4	NM_005359.3	0.374	0.0272	0.206	KEGG WNT Annotation
ERBB2	NM_001005862.1	-0.679	0.0319	0.222	EMTMetastasis
PKN1	NM_213560.1	0.357	0.0322	0.222	EMTMetastasis
PTGS2	NM_000963.1	-2.07	0.0335	0.222	Calcium Binding and Signaling, Cell Cycle
CXCL12	NM_000609.5	-3.36	0.0367	0.226	EMTMetastasis
BMP4	NM_001202.3	0.757	0.0372	0.226	Development & Differentiation
T	NM_003181.2	4.22	0.0383	0.226	Development & Differentiation, Transcription Factors
BAMBI	NM_012342.2	3.2	0.0408	0.226	Canonical Wnt Pathway
CCND1	NM_053056.2	-2.39	0.0425	0.226	Calcium Binding and Signaling, Cell Cycle, Development & Differentiation, KEGG WNT Annotation, WNT Signaling Negative Regulation, WNT Signaling Target Genes
FZD2	NM_001466.2	-2.36	0.0426	0.226	Calcium Binding and Signaling, Canonical Wnt Pathway, KEGG WNT Annotation
PRKACA	NM_002730.3	-0.319	0.0519	0.252	KEGG WNT Annotation
FOXN1	NM_003593.2	-3.47	0.055	0.252	Development & Differentiation
TCF7L1	NM_031283.1	-1.11	0.0575	0.252	Canonical Wnt Pathway, KEGG WNT Annotation, Transcription Factors
TBL1XR1	NM_024665.4	0.332	0.061	0.252	KEGG WNT Annotation
ETS2	NM_005239.4	0.771	0.0618	0.252	Transcription Factors
WNT10B	NM_003394.2	-1.67	0.0627	0.252	KEGG WNT Annotation
MYC	NM_002467.3	0.568	0.0639	0.252	Cell Cycle, KEGG WNT Annotation, Transcription Factors, WNT Signaling Target Genes

WNT2	NM_003391.2	-1.47	0.0647	0.252	Calcium Binding and Signaling, Canonical Wnt Pathway, KEGG WNT Annotation
FZD1	NM_003505.1	1.58	0.0668	0.252	Canonical Wnt Pathway, KEGG WNT Annotation
ZEB1	NM_001128128.1	0.351	0.0673	0.252	EMTMetastasis
SOX2	NM_003106.2	-2.25	0.0691	0.252	Cell Cycle, Development & Differentiation, Transcription Factors
POU5F1	NM_002701.4	-0.589	0.0704	0.252	Development & Differentiation, Transcription Factors
FN1	NM_212482.1	-2.03	0.0706	0.252	Development & Differentiation, Migration
FZD3	NM_017412.2	-1.04	0.0713	0.252	Canonical Wnt Pathway, KEGG WNT Annotation
FZD8	NM_031866.1	-3.06	0.0742	0.256	Canonical Wnt Pathway, KEGG WNT Annotation
TIMP1	NM_003254.2	-1.19	0.0766	0.259	EMTMetastasis
SERPINE1	NM_001165413.1	-2.58	0.0809	0.268	EMTMetastasis
PDGFRA	NM_006206.3	-1.69	0.0855	0.278	Development & Differentiation, Migration
APC	NM_000038.3	0.293	0.0896	0.28	Canonical Wnt Pathway, KEGG WNT Annotation, WNT Signaling Negative Regulation
RUVBL1	NM_003707.2	-0.333	0.0908	0.28	Canonical Wnt Pathway, KEGG WNT Annotation
RUNX2	NM_004348.3	-0.691	0.0945	0.284	Development & Differentiation, Transcription Factors
TCF4	NM_003199.1	0.362	0.105	0.309	Transcription Factors
LEF1	NM_016269.3	1.58	0.11	0.316	Canonical Wnt Pathway, KEGG WNT Annotation, Transcription Factors
CSNK1A1	NM_001892.4	0.219	0.111	0.316	Canonical Wnt Pathway, KEGG WNT Annotation
SOX9	NM_000346.2	0.811	0.119	0.326	Adhesion, Development & Differentiation, Transcription Factors
DVL1	NM_004421.2	0.306	0.127	0.339	Canonical Wnt Pathway, KEGG WNT Annotation, Planar Cell Polarity (PCP)
SMAD3	NM_005902.3	0.887	0.132	0.34	KEGG WNT Annotation

NRP1	NM_003873.5	-4.33	0.134	0.34	Development & Differentiation, Migration
PORCN	NM_022825.2	0.22	0.135	0.34	Canonical Wnt Pathway, KEGG WNT Annotation
DVL2	NM_004422.2	0.229	0.137	0.34	Canonical Wnt Pathway, KEGG WNT Annotation, Planar Cell Polarity (PCP)
AXIN2	NM_004655.3	2	0.14	0.343	Canonical Wnt Pathway, KEGG WNT Annotation, WNT Signaling Negative Regulation, WNT Signaling Target Genes
PRKACB	NM_182948.2	0.836	0.143	0.345	KEGG WNT Annotation
SNAI2	NM_003068.3	-2.53	0.145	0.345	EMTMetastasis
PPP3CB	NM_001142354.1	-0.129	0.15	0.351	KEGG WNT Annotation
KREMEN1	NM_001039570.1	-1.67	0.157	0.351	WNT Signaling Negative Regulation
SFRP1	NM_003012.3	-1.58	0.159	0.351	Canonical Wnt Pathway, KEGG WNT Annotation, WNT Signaling Negative Regulation
CTNNB1	NM_001098210.1	0.221	0.159	0.351	Canonical Wnt Pathway, KEGG WNT Annotation
DAB2	NM_001343.2	-2.05	0.16	0.351	Development & Differentiation, WNT Signaling Target Genes
WNT11	NM_004626.2	2.15	0.163	0.351	Calcium Binding and Signaling, KEGG WNT Annotation
NLK	NM_016231.2	0.267	0.164	0.351	KEGG WNT Annotation, WNT Signaling Negative Regulation
RAC1	NM_198829.1	0.213	0.168	0.351	KEGG WNT Annotation
CDKN2A	NM_000077.3	0.552	0.168	0.351	Cell Cycle, Development & Differentiation, Transcription Factors
CXXC4	NM_025212.1	-0.783	0.172	0.355	Canonical Wnt Pathway, KEGG WNT Annotation, WNT Signaling Negative Regulation
KLF5	NM_001730.3	1.01	0.195	0.392	Transcription Factors
PRKCG	NM_002739.3	-0.992	0.198	0.394	KEGG WNT Annotation
NANOG	NM_024865.2	-0.367	0.206	0.404	Development & Differentiation, Transcription Factors
IRS1	NM_005544.2	0.815	0.212	0.41	Migration

SKP1	NM_170679.2	-0.09	0.217	0.413	KEGG WNT Annotation
PPP3CA	NM_000944.4	0.758	0.218	0.413	KEGG WNT Annotation
RAC3	NM_005052.2	0.337	0.221	0.413	KEGG WNT Annotation
WNT4	NM_030761.3	-1.03	0.224	0.414	Calcium Binding and Signaling, Canonical Wnt Pathway, KEGG WNT Annotation
JUN	NM_002228.3	0.501	0.233	0.421	KEGG WNT Annotation, WNT Signaling Target Genes
TGFB3	NM_003239.2	0.398	0.254	0.455	Development & Differentiation
CCND3	NM_001760.2	0.342	0.279	0.493	KEGG WNT Annotation
MMP7	NM_002423.3	-1.71	0.293	0.502	Calcium Binding and Signaling, KEGG WNT Annotation, Proteolysis, WNT Signaling Target Genes
SIX1	NM_005982.3	-1.61	0.294	0.502	Development & Differentiation, Migration, Transcription Factors
PPARD	NM_006238.3	-0.146	0.299	0.506	Development & Differentiation, KEGG WNT Annotation, Migration, Transcription Factors, WNT Signaling Target Genes
FZD6	NM_003506.2	0.818	0.315	0.527	Canonical Wnt Pathway, KEGG WNT Annotation
IL6	NM_000600.1	-1.1	0.328	0.543	Development & Differentiation, Migration
VEGFA	NM_001025366.1	0.954	0.355	0.578	Development & Differentiation, Migration
AES	NM_001130.5	0.126	0.357	0.578	Canonical Wnt Pathway
WNT5A	NM_003392.3	-1	0.368	0.586	Calcium Binding and Signaling, KEGG WNT Annotation
WNT6	NM_006522.3	1.23	0.368	0.586	Calcium Binding and Signaling, Canonical Wnt Pathway, KEGG WNT Annotation
BIRC5	NM_001168.2	-0.393	0.376	0.587	Cell Cycle
CDH11	NM_001797.2	-1.44	0.377	0.587	EMTMetastasis

WNT7B	NM_058238.1	0.836	0.394	0.609	Calcium Binding and Signaling, Canonical Wnt Pathway, KEGG WNT Annotation
NRCAM	NM_005010.4	-0.83	0.405	0.62	Adhesion, Development & Differentiation, Migration
NKD1	NM_033119.3	-0.322	0.419	0.635	Canonical Wnt Pathway, KEGG WNT Annotation, Planar Cell Polarity (PCP), WNT Signaling Negative Regulation
FBXW4	NM_022039.3	-0.154	0.449	0.667	WNT Signaling Negative Regulation
CSNK2A1	NM_177559.2	0.0641	0.453	0.667	Canonical Wnt Pathway, KEGG WNT Annotation
FZD5	NM_003468.2	0.582	0.47	0.683	Canonical Wnt Pathway, KEGG WNT Annotation
LRP5	NM_002335.1	0.302	0.472	0.683	Canonical Wnt Pathway, KEGG WNT Annotation
FOSL1	NM_005438.2	-0.404	0.501	0.712	Cell Cycle, KEGG WNT Annotation, Transcription Factors, WNT Signaling Target Genes
SNAI1	NM_005985.2	0.24	0.569	0.797	EMTMetastasis
MYLK	NM_053032.2	-0.175	0.572	0.797	EMTMetastasis
CAMK2B	NM_001220.3	0.369	0.588	0.81	KEGG WNT Annotation
CTBP1	NM_001328.2	-0.0632	0.591	0.81	Canonical Wnt Pathway, KEGG WNT Annotation, WNT Signaling Negative Regulation
NFATC1	NM_172389.1	-0.36	0.601	0.812	Calcium Binding and Signaling, KEGG WNT Annotation
GPC4	NM_001448.2	1.71	0.602	0.812	Canonical Wnt Pathway
PRKCA	NM_002737.2	0.471	0.626	0.826	KEGG WNT Annotation
PLCB1	NM_182734.1	-0.534	0.629	0.826	KEGG WNT Annotation
DIXDC1	NM_001037954.3	-0.176	0.639	0.833	Canonical Wnt Pathway
EFNB1	NM_004429.4	0.197	0.654	0.845	Development & Differentiation, Migration
PPP3R1	NM_000945.3	0.0488	0.667	0.848	KEGG WNT Annotation
BCL9	NM_004326.2	-0.132	0.681	0.857	Canonical Wnt Pathway

EP300	NM_001429.2	0.057	0.688	0.857	Canonical Wnt Pathway, KEGG WNT Annotation
WNT2B	NM_024494.1	-0.256	0.696	0.857	Calcium Binding and Signaling, Canonical Wnt Pathway, KEGG WNT Annotation
MAPK8	NM_002750.2	0.117	0.707	0.857	KEGG WNT Annotation, Planar Cell Polarity (PCP)
JAG1	NM_000214.2	0.266	0.709	0.857	Development & Differentiation, Migration
ID2	NM_002166.4	-0.276	0.718	0.857	Cell Cycle, Development & Differentiation
PPP3CC	NM_005605.4	0.0829	0.722	0.857	KEGG WNT Annotation
SMO	NM_005631.3	-0.28	0.732	0.857	Development & Differentiation, Migration
GSK3B	NM_002093.2	0.0354	0.735	0.857	Canonical Wnt Pathway, KEGG WNT Annotation
ZEB2	NM_014795.3	0.207	0.735	0.857	EMTMetastasis
RHOA	NM_001664.2	-0.0369	0.737	0.857	KEGG WNT Annotation, Planar Cell Polarity (PCP)
CD44	NM_001001392.1	0.0591	0.739	0.857	Adhesion, Migration
FZD7	NM_003507.1	0.33	0.753	0.86	Canonical Wnt Pathway, KEGG WNT Annotation
CDH1	NM_004360.2	-0.306	0.756	0.86	Adhesion, Development & Differentiation, EMTMetastasis
EGFR	NM_201282.1	0.838	0.757	0.86	Adhesion, Calcium Binding and Signaling, Cell Cycle, Development & Differentiation, Migration
TGFB1	NM_000660.3	0.0283	0.812	0.903	EMTMetastasis
WNT5B	NM_032642.2	-0.0997	0.813	0.903	Calcium Binding and Signaling, KEGG WNT Annotation
FRZB	NM_001463.2	0.174	0.818	0.903	WNT Signaling Negative Regulation
CCND2	NM_001759.2	-0.0898	0.84	0.915	Cell Cycle, Development & Differentiation, KEGG WNT Annotation, WNT Signaling Target Genes
PPAP2B	NM_003713.3	-0.259	0.856	0.926	Migration
EGR1	NM_001964.2	-0.149	0.881	0.928	Development & Differentiation, Transcription Factors

TCF7	NM_003202.2	0.0397	0.883	0.928	Canonical Wnt Pathway, KEGG WNT Annotation, Transcription Factors
AHR	NM_001621.3	-0.13	0.885	0.928	Cell Cycle, Transcription Factors
VANGL1	NM_138959.2	0.091	0.886	0.928	KEGG WNT Annotation
BTRC	NM_033637.2	-0.0186	0.887	0.928	KEGG WNT Annotation, WNT Signaling Negative Regulation, WNT Signaling Target Genes
LRP6	NM_002336.1	0.0948	0.904	0.939	Canonical Wnt Pathway, KEGG WNT Annotation, WNT Signaling Negative Regulation
PRKX	NM_005044.1	-0.0233	0.916	0.946	KEGG WNT Annotation
RAC2	NM_002872.3	-0.0121	0.928	0.951	KEGG WNT Annotation
SFRP4	NM_003014.2	-0.106	0.935	0.953	Canonical Wnt Pathway, KEGG WNT Annotation, WNT Signaling Negative Regulation
STAT3	NM_139276.2	-0.0278	0.943	0.955	EMTMetastasis
CXCR4	NM_003467.2	-0.0396	0.96	0.966	EMTMetastasis
WNT3	NM_030753.3	0.0117	0.985	0.985	Calcium Binding and Signaling, Canonical Wnt Pathway, KEGG WNT Annotation
**GENES BELOW THE BACKGROUND LEVEL**					
CTGF	NM_001901.2	<20 counts			
PRKACG	NM_002732.2	<20 counts			
DPP10	NM_020868.3	<20 counts			
WNT1	NM_005430.2	<20 counts			
TWIST1	NM_000474.3	<20 counts			
FGF4	NM_002007.2	<20 counts			
MMP3	NM_002422.3	<20 counts			

IGF2	NM_001127598.1	<20 counts			
WNT8A	NM_058244.2	<20 counts			
PPP3R2	NM_147180.2	<20 counts			
TWIST2	NM_057179.2	<20 counts			
PROM1	NM_006017.1	<20 counts			
PLCB4	NM_000933.3	<20 counts			
WNT9A	NM_003395.1	<20 counts			
DKK2	NM_014421.2	<20 counts			
DKK1	NM_012242.2	<20 counts			
WISP1	NM_080838.1	<20 counts			
WNT8B	NM_003393.2	<20 counts			
FZD10	NM_007197.2	<20 counts			
FZD4	NM_012193.2	<20 counts			
WNT7A	NM_004625.3	<20 counts			
SOST	NM_025237.2	<20 counts			
KCNQ1	NM_181798.1	<20 counts			
SFRP2	NM_003013.2	<20 counts			
DLK1	NM_003836.4	<20 counts			
WNT16	NM_057168.1	<20 counts			
FGF7	NM_004675.2	<20 counts			
TLE1	NM_005077.3	<20 counts			
WIF1	NM_007191.2	<20 counts			

COL1A2	NM_000089.3	<20 counts			
DKK3	NM_001018057.1	<20 counts			
WNT9B	NM_003396.1	<20 counts			
FZD9	NM_003508.2	<20 counts			
PYGO1	NM_015617.1	<20 counts			
**HOUSEKEEPING IN THE PANEL**					
CC2D1B	NM_032449.2	Housekeeping			
COG7	NM_153603.3	Housekeeping			
EDC3	NM_001142443.1	Housekeeping			
GPATCH3	NM_022078.2	Housekeeping			
HDAC3	NM_003883.2	Housekeeping			
MTMR14	NM_022485.3	Housekeeping			
NUBP1	NM_001278506.1	Housekeeping			
PRPF38A	NM_032864.3	Housekeeping			
SAP130	NM_024545.3	Housekeeping			
SF3A3	NM_006802.2	Housekeeping			
TLK2	NM_006852.2	Housekeeping			
ZC3H14	NM_001160103.1	Housekeeping			
